# Plasma TF activity predicts cardiovascular mortality in patients with acute myocardial infarction

**DOI:** 10.1186/1477-9560-7-11

**Published:** 2009-07-02

**Authors:** Birgit A Steppich, Siegmund Lorenz Braun, Andreas Stein, Gabriele Demetz, Philip Groha, Albert Schömig, Nicolas von Beckerath, Adnan Kastrati, Ilka Ott

**Affiliations:** 1 Medizinische Klinik, Klinikum rechts der Isar der Technischen Universität München, , Munich, Germany; 2 Klinik für Kardiologie, Deutsches Herzzentrum München der Technischen Universität, Munich, Germany; 3 Institut für Laboratoriumsmedizin, Deutsches Herzzentrum München der Technischen Universität, Munich, Germany

## Abstract

**Objectives and Background:**

Tissue factor (TF) contributes to thrombosis following plaque disruption in acute coronary syndromes (ACS). Aim of the study was to investigate the impact of plasma TF activity on prognosis in patients with ACS.

**Methods and Results:**

One-hundred seventy-four patients with unstable Angina pectoris (uAP) and 112 patients with acute myocardial infarction (AMI) were included with a mean follow up time of 3.26 years. On admission, plasma TF activity was assessed. Patients were categorized into 2 groups: a high-TF activity group with TF >24 pmol/L and low TF activity group with TF ≤ 24 pmol/L. Fifteen cardiovascular deaths occurred in the uAP group and 16 in the AMI group. In AMI TF activity was 24,9 ± 2,78 pmol/l (mean ± SEM) in survivors and 40,9 ± 7,96 pmol/l in nonsurvivors (P = 0.024). In uAP no differences were observed (25.0 ± 8.04 pmol/L nonsurvivors vs. 25.7 ± 2.14 pmol/L survivors; P = 0.586). Kaplan-Meier estimates of survival at 3.26 years regarding TF activity in AMI were 81.3% and 92.2% with an hazard ratio of 3.02 (95% CI [1.05–8.79], P = 0.03). The Cox proportional hazards model adjusting for correlates of age and risk factors showed that plasma TF activity was an independent correlate of survival (hazard ratio 9.27, 95% CI [1.24–69.12], P = 0.03). In an additional group of patients with uAP and AMI, we identified circulating microparticles as the prevailing reservoir of plasma TF activity in acute coronary syndromes.

**Conclusion:**

Systemic TF activity in AMI has an unfavorable prognostic value and as a marker for dysregulated coagulation may add to predict the atherothrombotic risk.

## Introduction

Atherothrombosis, characterized by a disruption of atherosclerotic lesions superimposed with thrombus formation, is the major cause of acute coronary syndromes (ACS) and cardiovascular death. Tissue factor (TF) as the cellular initiator of the extrinsic coagulation cascade plays a key role in intravascular thrombus formation by allosterically activating factor VII (FVII). Over the last decades it has been shown that, in addition to intravascular TF, circulating TF is implicated in arterial and venous thrombosis [1-5]. Current studies have shown that plasma TF is comprised of: a) TF associated with microparticles (MP) and b) an alternatively spliced TF variant that lacks the transmembrane domain [6]. In living mice this plasma or blood-borne TF accumulates in newly formed thrombi via monocyte-derived MP and contributes to thrombus growth and propagation [2,7]. Several studies have demonstrated increased levels of circulating TF in patients with unstable Angina pectoris (uAP) and acute myocardial infarction (AMI) [8-12]. Therefore, it has long been speculated that, in cases with no plaque rupture or only fractional superficial erosion, thrombus formation may depend on circulating levels of TF. Consistent with this idea, several studies suggest that the levels of circulating TF and other haemostatic biomarkers may correlate to adverse cardiovascular events and mortality in patients with ACS [13-17]. However, all these data are based on immunoreactive measurements and provide no evidence that circulating TF in ACS is functional or is capable of contributing to systemic hypercoagulability. To date, data addressing plasma TF activity are lacking. Since TF antigen measurements do not necessarily reflect the functional capacity and integrity of TF, measuring TF activity might be a better approach for stratifying cardiovascular risk. Therefore, aim of the present study was to investigate plasma TF activity in patients with ACS and evaluate its capacity to predict future cardiovascular and overall mortality.

## Methods

### Study population

This prospective study enrolled 286 consecutive patients that were referred to the hospital between July 2001 and February 2002 for acute coronary syndromes. The diagnosis of AMI was established by the presence of chest pain lasting >20 minutes associated with electrocardiographic and enzymatic changes (Peak CK ≥ 300 U/l). Electrocardiographic changes included: an ST-segment elevation of at least 0.1 mV in two or more limb leads, an elevation of at least 2 mV in two or more contiguous precordial leads on the surface electrocardiogram, or a new-onset left bundle branch block. Unstable Angina was defined as a crescendo pattern of chest pain at rest with documented non-specific electrocardiographic changes including: a transient ST segment depression or elevation of at least 0.1 mV in at least 2 contiguous electrocardiographic leads or a T wave inversion. Patients with interfering non-cardiac diseases and malignancies were not included in the study. On admission, blood was drawn from all subjects under standardized conditions and plasma samples were stored at -80°C until analysis. All patients underwent urgent coronary angiography for percutaneous coronary intervention (PCI). The study protocol was approved by the institutional ethics committee and informed consent was obtained from all subjects.

To analyze the sources of circulating TF activity, an additional group of 36 consecutive patients with uAP and AMI were recruited between December 2005 and November 2006. A control group comprised 28 patients with stable angina (sAP) undergoing elective PCI.

### Biochemical and microparticle analysis

Plasma TF activity was measured with an FXa generation assay (Actichrome TF, American Diagnostica; Detection treshold 2 pM, intra-assay variabilities <10%; inter-assay variabilities <10%). To inhibit endogenous TFPI TF activity was measured using an FXa generation assay in the absence and presence of 100 μg/ml rabbit anti-TFPI-1 IgG (kindly provided by Dr. W. Ruf) or nonimmune rabbit IgG (Sigma). TF activity in the presence of anti-TFPI mAbs represents the total TF activity in the plasma. TF activity associated with circulating MP was measured by first isolating MP by antibody capture and then analyzing FXa generation using a commercial available assay (Actichrome^®^Microparticle activity #817, American diagnostic; Detection treshold ≤ 0,05 nM, intra-assay variabilities 3–8%; inter-assay variabilities 5–10%). Plasma TF antigen was measured by immunoassay (Imubind Tissue Factor ELISA kit, American Diagnostica; Detection treshold 1,4 pM, intra-assay variabilities 4,5%; inter-assay variabilities 7,5%).

Serum concentrations of creatine kinase, C-reactive protein (CRP), fibrinogen, and brain natriuretic protein (BNP) (Roche NT-pro-BNP sandwich electrochemiluminescent assay, Roche Diagnostics, Mannheim, Germany) were determined in routine clinical chemistry laboratory analyses.

For analysis of circulating MP, platelet free plasma (PFP) was immediately snap frozen in liquid nitrogen and stored at -80°C until use. To separate MP from PFP, aliquots of 250 μl were centrifuged for 60 min at 100,000 × g; then 225 μl of the supernatant was decanted and stored for further analysis. MP were resuspended in the remaining 25 μl. Flowcytometric analyses of MP suspensions were performed according to the manufacturer's instructions using annexin V Alexa Fluor^® ^568 in an appropriate buffer (Boehringer Mannheim, Germany) and either specific monoclonal antibodies (mAb) or isotype-matched control antibodies. Antibodies were labeled with fluoresceinisothiocyonate (FITC), peridinin-chlorophyll-protein complex (PerCP), or Allophycocyanin (APC) and included: FITC-conjugated anti-TF antibody (American diagnostic), CD41 PerCP (Coulter), and CD 66 APC (BectonDickinson). Before FACS analysis was performed, a defined number of latex beads (LB30, Sigma) were added to the samples in order to calculate the absolute amount of MPs. Analysis was stopped when 5000 beads were counted.

### Clinical follow-up and endpoint definition

All enrolled patients were contacted for follow-up with a mean of 3.26 years after the percutaneous coronary intervention. The primary outcome measures of the study were: cardiovascular mortality, which included a) fatal myocardial infarction and b) ventricular arrhythmias with heart failure as a consequence of MI, and all cause mortality.

### Statistical analysis

Discrete variables were expressed as counts (%) and compared by chi-square or Fisher's exact test. Continuous variables were expressed as mean ± S.D. and compared by the Wilcoxon's rank sum test. The Youden-index was used to determine statistically reliable cut-off value of TF by maximizing the sum of sensitivity and specificity in reference to observed survival state. In this term, area under the ROC-curve (AUC) was reported to assess overall prognostic performance of plasma TF activity. Further, the corresponding 95% confidence interval of AUC was reported indicating statistically significant predictive capability if the critical value of 0.5 is not included. Spearman-Rho correlation coefficient was used to identify correlates of TF activity. Survival analysis was performed by applying the Kaplan-Meier method. Differences in survival were assessed with log rank test. Cox's proportional hazard model was used to assess the independent association of TF activity with mortality. All statistical analyses were performed using SPSS 15.0. P-values < 0.05 were considered statistically significant.

## Results

### Baseline characteristics

Of the 286 patients enrolled in the study, 112 presented with AMI and 174 were classified as uAP. During the follow up 16 cardiovascular deaths and 8 non-cardiovascular deaths were reported among patients with AMI; similarly, 15 cardiovascular deaths and 9 non-cardiovascular deaths were reported among patients with uAP. In AMI the majority of cardiovascular death occurred in the first year caused by cardiogenic shock or terminal heart failure, whereas in uAP only 3 deaths occurred during this time. The majority of cardiovascular death in uAP occurred after 1 year or later due to reinfarction, terminal heart failure and sudden cardiac death. According to a receiver operating characteristic (ROC) analysis patients were categorized into 2 groups: the high-TF activity group included patients with TF values >24 pmol/L, the low TF activity group included patients with TF values ≤ 24 pmol/L. ROC – analysis revealed a substantial prognostic capability of TF with an AUC of 0.66 (95%CI: 0.51 to 0.81). The clinical characteristics of each patient group and the incidence of cardiovascular death are shown in Table 1 and 2. As expected, patients with AMI that died from cardiovascular causes were older and had higher peak CK and NT-proBNP levels at baseline than those that survived (data not shown). Also, on admission they had a reduced left ventricular ejection fraction and presented with a higher Killip class. Patients with uAP that died had a higher prevalence of hypertension and preclinical resuscitation than those that survived. No other characteristics were different between patients that died and those that survived. The majority of AMI patients received abciximab, and the majority of uAP patients received only heparin.

**Table 1:** Baseline characteristics of patients with acute myocardial infarction (n = 112)

Characteristic	TF activity ≤ 24 pmol (n = 64)	TF activity > 24 pmol (n = 48)	P
TF Activity, (pmol)	7.0 ± 0.90	54.2 ± 3.30	<0.001
TF Activity + anti TFPI, (pmol)	39.7 ± 2.25	65.8 ± 2.99	<0.001

*Demographics*			
Median age, years	64.5 ± 1.54	58.7 ± 1.95	0.02
Male sex	54 (84.4%)	37 (77.1%)	1.0
Number of CV death (n)	5 (7.8%)	11 (22.9%)	0.02
Time to death (days)	437.3 ± 145.72	218.0 ± 55.63	0.68
Days of follow up (days)	1460.0 ± 0.0	1460.0 ± 0.0	1.0
*Pevious medical history*			
Myocardial infarction	15 (24.4%)	8 (16.7%)	0.005
ACVB	5 (7.8%)	2 (4.2%)	0.001
*Cardiovascular risk factors*			
Arterial hypertension	40 (62.5%)	34 (70.8%)	0.17
Diabetes mellitus	14 (21.9%)	15 (31.3%)	0.001
Hypercholesterolemia	39 (60.9%)	25 (52.1%)	0.41
Current smoker	42 (65.6%)	36 (75.0%)	0.001
*Biochemical markers*			
Peak creatine kinase, U/l	1475.0 ± 357.91	1897.7 ± 276.5	0.03
C-reactive protein, mg/dl	44.7 ± 11.22	26.2 ± 6.79	0.25
NT-pro-BNP, pg/ml	1739.4 ± 402.93	3946.8 ± 1442.53	0.28
*Clinical data*			
Prehospital CPR	5 (7.8%)	10 (20.8%)	0.05
Cardiogenic shock	9 (14.1%)	13 (27.1%)	0.09
Killip's class			
I	47 (73.4%)	25 (52.1%)	0.04
II	8 (12.5%)	10 (20.8%)	0.09
III	3 (4.7%)	6 (12.5%)	0.60
IV	6 (9.4%)	7 (14.6%)	0.40
*Angiographic data*			
Left ventricular function	46.2 ± 1.56	45.3 ± 1.97	0.77
Target vessel			
LAD	27 (42.2%)	29 (60.4%)	0.30
RCA	30 (46.9%)	12 (25.0%)	0.03
LCx	7 (10.9%)	7 (14.6%)	0.57
Extent of coronary artery			
Disease			
1-vessel-disease	18 (28.1%)	15 (31.3%)	0.72
2-vessel-disease	18 (28.1%)	14 (29.2%)	0.29
3-vessel-disease	28 (43.8%)	19 (39.6%)	0.16
Medication on admission			
Aspirin	64 (100%)	48 (100%)	1.0
Heparin	12 (18.8%)	4 (8.3%)	0.07
Clopidogrel	54 (84.4%)	44 (91.7%)	0.83
Beta-Blockers	18 (28.1%)	10 (20.8%)	0.07
ACE-Inhibitors	10 (15.6%)	7 (14.6%)	0.86
Statins	12 (18.8%)	5 (10.4%)	0.85

ACVB: aortocoronary bypass grafting; CPR: cardiopulmonary resuscitation

**Table 2:** Baseline characteristics of patients with unstable Angina pectoris (n = 174)

Characteristic	TF activity ≤ 24 pmol (n = 106)	TF activity > 24 pmol (n = 68)	P
TF Activity, (pmol)	7.39 ± 0.60	53.9 ± 2.76	<0.001
TF Activity + anti TFPI, (pmol)	41.36 ± 1.92	66.17 ± 2.38	<0.001

*Demographics*			
Median age, years	66.6 ± 1.05	67.2 ± 1.36	0.79
Male sex	76 (71.7%)	37 (54.4%)	0.88
Number of CV death (n)	10 (9.4%)	5 (7.4%)	
Time to death (days)	655.47 ± 123.49	642.78 ± 137.90	0.93
Days of follow up (days)	1414.48 ± 11.24	1443.75 ± 8.39	0.13
*Previous medical history*			
Myocardial infarction	35 (33.0%)	21 (30.9%)	0.74
ACVB	13 (12.3%)	7 (10.3%)	0.69
*Cardiovascular risk factors*			
Arterial hypertension	91 (85.9%)	56 (82.4%)	0.54
Diabetes mellitus	28 (26.4%)	28 (41.2%)	0.07
Hypercholesterolemia	69 (65.1%)	40 (58.8%)	0.35
Current smoker	51 (48.1%)	30 (44.1%)	0.50
*Biochemical markers*			
Peak creatine kinase, U/l	92.7 ± 5.39	188.2 ± 15.39	0.56
C-reactive protein, mg/dl	22.9 ± 4.00	24.8 ± 4.37	0.19
NT-pro-BNP, pg/ml	2321.24 ± 490.50	2176.1 ± 306.79	0.13
*Clinical data*			
Prehospital CPR	1 (0.9%)	1 (1.5%)	0.75
Cardiogenic shock	2 (1.9%)	0 (0.0%)	0.26
Killip's class			
I	103 (97.2%)	64 (94.1%)	0.32
II	2 (1.9%)	1 (1.5%)	0.84
III	0 (0.0%)	2 (2.9%)	0.08
IV	1 (0.9%)	1 (1.5.%)	0.75
*Angiographic data*			
Left ventricular function	52.6 ± 1.38	51.5 ± 1.77	0.67
Target vessel			
LAD	44 (41.5%)	33 (48.5%)	0.27
RCA	33 (31.1%)	24 (35.3%)	0.20
LCx	29 (27.4%)	11 (16.8%)	0.14
Extent of coronary artery			
Disease			
1-vessel-disease	21 (19.8%)	13 (19.1%)	0.90
2-vessel-disease	32 (30.2%)	21 (30.9%)	0.76
3-vessel-disease	53 (50.0%)	34 (50.0%)	0.85
Medication on admission			
Aspirin	106 (100%)	68 (100.0%)	1.00
Heparin	63 (63.4%)	38 (55.9%)	0.79
Clopidogrel	91 (85.8%)	49 (72.1%)	0.54
Beta-Blockers	62 (58.5%)	38 (55.8%)	0.82
ACE-Inhibitors	53 (50.0%)	29 (42.7%)	0.34
Statins	44 (41.5%)	24 (35.3%)	0.46

ACVB: aorto-coronary bypass crafting; CPR: cardiopulmonary resuscitaion

### TF activity and cardiovascular mortality

**Figure 1:**
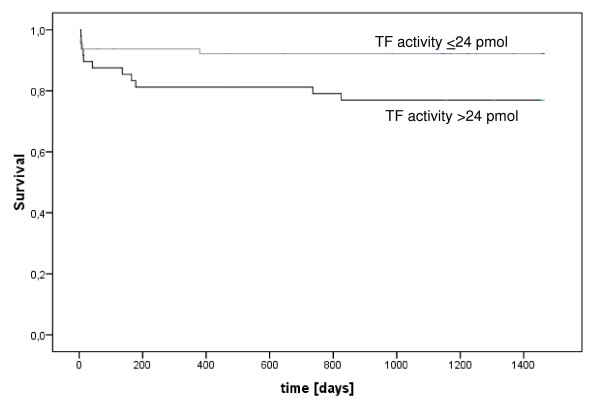
**Kaplan-Meier cardiovascular mortality curves according to low and high TF activity in AMI**. Patients with AMI were divided in a group with high (> 24 pmol/L) and a group with low plasma TF activities on admission (≤ 24 pmol/L). (p = 0,03).

In patients with AMI the TF activity was 24.9+2.78 pmol/L (mean+SEM) in survivors and 40.9+7.96 pmol/L in nonsurvivors (P = 0.024). Kaplan-Meier estimates of survival regarding TF activity in AMI above or below 24 pmol were 81.3% and 92.2% with an odds ratio of 3.02 (95% CI [1.05–8.79], P = 0.03; Figure 1). In uAP no differences in TF activity were observed in survivors and nonsurvivors (25.7 ± 2.14 pmol/L versus 25.0 ± 8.04 pmol/L; P = 0.59). The Cox proportional hazards model adjusting for correlates of mortality showed that plasma TF activity was a prognostic factor of mortality in patients with AMI (Table 3).

**Table 3:** Cox regression analysis for cardiovascular mortality in AMI patients in relation to risk factors

	Significance	Exp(B)	95%CI lower	95%CI upper
TF activity	0.03	9.27	1.24	69.12
Age	0.01	1.07	1.01	1.13
Hypertension	0.72	0.60	0.04	9.83
Diabetes	0.96	1.06	0.19	5.84
Smoking	0.96	0.97	0.31	3.03
Cholesterol	0.79	1.46	0.09	24.44

### Relationships between TF activity, inflammation, and necrosis

Among patients with AMI, TF activity correlated with parameters of myocardial necrosis (peak CK: R = 0.22, *P *= 0.025; peak CK-MB: R = 0.23; *P *= 0.019; CK on admission: R = 0.23, *P *= 0.037), Killip class (R = 0.22, *P *= 0.022), and incidence of cardiopulmonary resuscitation on admission (R = 0.20, *P *= 0.037). No association was found between TF activity and markers of inflammation. In contrast, among patients with uAP, TF activity was only correlated with parameters of inflammation (CRP: R = 0.17, *P *= 0.03; fibrinogen: R = 0.23, *P *= 0.005, and NT-proBNP: R = 0.16, *P *= 0.035) but not with parameters of myocardial necrosis, Killip class, or incidence of cardiopulmonary resuscitation.

### Sources of Circulating TF

To evaluate the functional significance and the underlying sources of circulating TF, we performed a second study comparing 36 patients with uAP and AMI and 28 control patients with sAP. In contrast to patients with ongoing ACS, almost no TF activity was detected in the circulation of sAP patients (0.0 ± 0.0 pmol vs 0.5 ± 0.16 pmol, *P *= 0.008, Table 4), even after abrogating the endogenous inhibition by TFPI-1 (0.98 ± 0.4 pmol vs 12.63 ± 1.82 pmol, *P *< 0.001).

**Table 4:** Coagulation markers in patients with stable Angina pectoris and an additional population of patients with ACS

	sAP (n = 28)	ACS (n = 36)	p
TF activity (pmol)	0.0 ± 0.0	0.5 ± 0.16	0.008
TF activity + anti-TFPI Ab (pmol)	0.98 ± 0.4	12.63 ± 1.82	<0.001
MP TF activity (nM)	0.24 ± 0.04	0.39 ± 0.07	0.03

**Figure 2:**
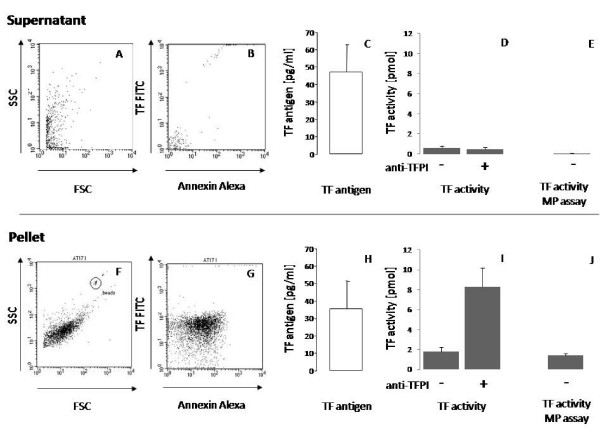
**Differential analysis of soluble TF (top row) and microparticle-associated TF (bottom row) in human plasma**. In both supernatant and pellet fractions, the frequencies of TF+Annexin+ microparticles were analyzed by FACS analysis. Representative dot blots of a patient with ACS are shown with (A, F) light-scatter-staining, and (B, G) specific-staining. (C, H) Concentrations of TF antigen were measured by immunoassay. (D, I) Plasma TF activity with or without (+/-) anti-TFPI-1 was measured by FXa generation assay. (E, J) Specific microparticle-associated TF activity was measured following antibody capture of microparticles. Values are expressed as mean ± SEM.

Currently there are two known sources that contribute to circulating TF: alternatively spliced soluble TF (asTF) and MP bearing membrane-bound TF. In order to analyze both fractions we applied a differential centrifugation procedure, resulting in pellet and supernatant fractions. FACS analysis showed the pellet was enriched in MP, while no viable cell particles were found in the supernatant (Figure 2A, B, F, G). Nevertheless, both fractions contained comparable amounts of TF antigen (Figure 2C, H). We reasoned that the pellet fraction most likely represented circulating MPs bearing TF, and the supernatant fraction contained a soluble form of TF. The pellet fraction exhibited a clear TF activity, which further increased after inhibition of endogenous TFPI. However, the supernatant had only little TF activity, devoid of any regulation by TFPI (Figure 2D, I). No TF activity was detected in the supernatant using the assay for MP-bound TF activity (Figure 2E, J). In the pellet, TF activity measured by the chromogenic and the MP assays were comparable, indicating that MPs were a main component of plasma TF activity. In contrast, in the supernatant, soluble forms of TF contributed to circulating TF antigen, but possessed no TF activity and were not regulated by TFPI. Flow cytometric characterization of the MPs of patients with ACS revealed that circulating MPs stained double-positive for platelet (CD41) and granulocyte (CD66b) markers (Table 5). Among the MPs that were Annexin+TF+, the percentages that were also CD41+CD66b+ correlated with the basal TF activity. Thus, in the plasma of patients with ACS, the TF activity was a consequence of circulating MPs.

**Table 5:** Number and cellular origin of circulating Microparticles in patients with ACS (n = 22)

	ACS (n = 22)
Annexin+ Microparticles, (10^3^/μl)	9.7 ± 3.85
%TF positive	56.8 ± 5.01
%CD41 positive	13.4 ± 2.19
%CD66 positive	15.0 ± 3.22
%CD66/CD41 double positive	49.2 ± 6.4
Annexin+ TF+ Microparticles, (10^3^/μl)	5.2 ± 2.26
%CD41 positive	10.6 ± 1.65
%CD66 positive	9.8 ± 3.22
%CD66/CD41 double positive	64.5 ± 5.00

## Discussion

This study shows that, the baseline plasma TF activity was an independent predictor for cardiovascular death in patients with AMI; furthermore, plasma TF activity was correlated to parameters of myocardial necrosis. In contrast, in patients with uAP, circulating TF activity was not related to the long term prognosis.

This is the first study to address the issue of whether circulating TF activity has an impact on cardiovascular mortality in patients with ACS. The associations between TF antigen and cardiovascular risk have been extensively studied with conflicting results. While some studies involving ACS patients reported no association between plasma TF levels and outcomes [18,19], others found TF antigen to be an independent predictor of cardiovascular events [12,14,16,17]. However TF antigen levels may not adequately reflect the thrombogenic potential of circulating TF, [20,21]. Although we found a significant correlation between TF antigen and TF activity (data not shown), the indicative role of TF activity showed differences among subpopulations. In patients with AMI, plasma TF activity was significantly increased in those that died compared to those that survived, while in patients with uAP no such association was observed.

Sources of plasma TF include MP-associated TF, truncated degradation products of TF, and the recently identified alternatively spliced TF variant that lacks a transmembrane-domain [4]. Herein we demonstrate that plasma membrane-bound TF was procoagulant, whereas soluble TF was inactive and that circulating MPs constitute the main reservoir of plasma TF activity. MPs have been shown to emerge from atherosclerotic plaques, leukocytes, platelets [22] and smooth muscle cells [23]. In AMI TF activity was associated with markers of myocardial necrosis. Since cardiomyocytes constitutively express TF and release TF positive procoagulant MP under inflammatory conditions, it is reasonable to speculate that MPs bearing TF in ongoing AMI may arise from necrotic myocardium [24,25]. MPs not only represent reliable hallmarks of cell damage, but also influence endothelial dysfunction, inflammation, and leukocyte adhesion [26,27]. Consistent with previous studies TF activity in the control group was below the assay's detection limit of 0.1 pM. [28]. The increase in TF activity observed in the present study, might reflect a systemic hypercoagulable state [29,30]. There is growing evidence that circulating TF is potentially active in an encrypted state, where it can bind FVII, but lacks proteolytic activity [31]. During thrombosis, encrypted TF can become available [1]. Among the mechanisms that control encryption and decryption phosphatidyl serine exposure, calcium influx, lipid raft dissociation, and Cys^186^–Cys^209 ^disulfide formation appear to play a role [21,31-33]. The significance of the present findings is supported by the fact that TF activity was an independent predictor for cardiovascular mortality in AMI even after adjustment for possible confounders. In accordance recent studies demonstrate, that MP linked tissue factor activity is associated to fibrinolysis failure in ST segment elevation infarction and is significantly elevated in the occluded coronary artery of STEMI patients [34,35]. Although the clinical relevance of circulating TF activity is still a matter of debate, our data together with these publications underline the pathophysiological relevance of circulating TF in coronary atherothrombosis. However, it has to be stressed that the small sample size limits the study due to low incidence of deaths during the follow-up.

Our data corroborate the hypothesis that circulating TF presents a high risk blood condition that is prone to thrombosis. Therefore the plasma TF activity may serve as a marker for dysregulated coagulation to predict atherothrombotic risk. Furthermore, pharmacological control of plasma TF activity might reduce atherothrombotic risk.

## Competing interests

The authors declare that they have no competing interests.

## Authors' contributions

BAS participated in the design and coordination of the study, collected and assembled the data, carried out the flow cytometric and microparticle analyses and drafted the manuscript. SLB carried out biochemical analyses. AS, GD and PG collected and assembled the data. AS, NvB and AK participated in the study design, the discussion of study data and the revision of the manuscript. IO conceived the study, performed the statistical analysis and revised the manuscript. All authors read and approved the final manuscript
